# Efficacy and safety of vismodegib in periocular and orbital basal cell carcinoma: a systematic review and meta-analysis

**DOI:** 10.3389/fmed.2026.1722574

**Published:** 2026-05-11

**Authors:** Dalal Alessa

**Affiliations:** Department of Ophthalmology, College of Medicine - Imam Mohammad Ibn Saud Islamic University (IMSIU), Riyadh, Saudi Arabia

**Keywords:** adverse events, basal cell carcinoma, exenteration, hedgehog inhibitor, orbital, periocular, recurrence, vismodegib

## Abstract

**Background:**

Periocular and orbital basal cell carcinoma (BCC) represents a therapeutic challenge due to its proximity to critical structures, the risk of disfigurement, and the potential need for exenteration in advanced disease. Vismodegib, a Hedgehog pathway inhibitor, offers a non-surgical alternative, but its overall efficacy and tolerability in this subset remain unclear.

**Aim:**

This systematic review and meta-analysis aimed to evaluate the efficacy, safety, and recurrence outcomes of vismodegib in periocular and orbital BCC.

**Methods:**

A systematic search of PubMed, Scopus, and Web of Science was conducted. Eligible studies included prospective or retrospective cohorts and case series with ≥7 patients treated with vismodegib. Primary outcomes were complete and partial response rates; secondary outcomes included recurrence, exenteration, and adverse events. Pooled incidence rates with 95% confidence intervals (CI) were calculated using a random-effects model.

**Results:**

Eighteen studies, with 764 patients, were included. The pooled complete response rate was 38.4% (95% CI: 36.6–57.4), and partial response occurred in 39.2% (95% CI: 32.4–47.5). Recurrence following discontinuation was reported in 17.4% (95% CI: 7.0–30.3). Despite vismodegib therapy, exenteration was performed in 28.1% (95% CI: 12.3–34.5). Adverse events were almost universal (90.3, 95% CI: 81.0–94.8), with alopecia (57.1%), muscle spasms (65.8%), decreased appetite/anorexia (28.2%), and weight loss (38.2%) being the most frequent. Severe adverse events (grade ≥3) occurred in 25.5% of patients, and treatment discontinuation due to toxicity was noted in 31.2%.

**Conclusion:**

Vismodegib provides meaningful tumor regression in periocular and orbital BCC, enabling globe preservation in a subset of patients and reducing the need for exenteration. However, high recurrence rates after discontinuation and the substantial burden of adverse events limit long-term tolerability. Careful patient selection and close monitoring are essential for optimizing outcomes.

## 1 Introduction

Basal cell carcinoma (BCC) is the most common malignant tumor of the periocular region, accounting for nearly 90% of eyelid malignancies worldwide (1, 2). BCC represents the most common human malignancy worldwide, with incidence rates continuing to rise in many regions due to aging populations, cumulative ultraviolet exposure, and improved detection. Although periocular tumors account for a relatively small proportion of all cutaneous BCCs, they represent the vast majority of malignant eyelid tumors and are associated with disproportionate morbidity because of their proximity to critical ocular and orbital structures (1, 2). Its slow growth and low metastatic potential often make it a highly curable disease when diagnosed early and managed appropriately (3, 4). Surgical excision with histologically controlled margins, either through standard wide local excision or Mohs micrographic surgery, remains the gold standard of treatment, offering cure rates exceeding 95% (5, 6). However, despite its generally indolent course, BCC arising in the periocular area presents unique challenges. The proximity to critical structures such as the orbit, lacrimal system, and globe means that complete excision can be technically difficult (7). In some cases, surgery carries the risk of significant cosmetic deformity or functional impairment, and in more advanced tumors, exenteration may be the only option (8).

Periocular BCCs can behave more aggressively than lesions in other anatomic sites (9, 10). Histological subtypes such as morpheiform, infiltrative, or micronodular BCC are associated with higher recurrence rates and deeper tissue invasion (11). Recurrent tumors after prior surgery or radiotherapy are also notoriously difficult to control (12). The management of these cases is particularly challenging when patients are elderly, medically frail, or unwilling to undergo mutilating procedures (13). Orbital involvement, which is seen in a minority of cases, further complicates management and often necessitates exenteration, a procedure that carries profound psychological and functional consequences (14).

The molecular pathogenesis of BCC has been closely linked to dysregulation of the Hedgehog (Hh) signaling pathway, which plays a key role in cell differentiation and tissue homeostasis during embryogenesis (15, 16). Aberrant activation of the pathway, typically through mutations in PTCH1 or SMO genes, leads to uncontrolled cellular proliferation and tumorigenesis (17). This discovery provided the rationale for developing targeted inhibitors of Hedgehog signaling. Vismodegib, the first-in-class oral Hh pathway inhibitor, was approved by the US Food and Drug Administration in 2012 for the treatment of adults with locally advanced BCC (laBCC) or metastatic BCC (mBCC) who are not candidates for surgery or radiotherapy (18, 19). By binding to and inhibiting Smoothened (SMO), a key transmembrane protein in the Hh cascade, vismodegib effectively suppresses downstream signaling, halting tumor growth and often inducing regression (20).

Since its approval, vismodegib has demonstrated clinical efficacy across multiple settings, particularly in patients with advanced disease where surgical or radiotherapeutic options are limited (21). Clinical trials, such as the pivotal ERIVANCE study, established its role in achieving meaningful tumor responses, with durable disease control in a subset of patients (22). Vismodegib, the first-in-class oral Hh pathway inhibitor, was approved by the US Food and Drug Administration in 2012 for the treatment of adults with locally advanced BCC (laBCC) or metastatic BCC (mBCC) who are not candidates for surgery or radiotherapy (21). However, most of the evidence has focused on cutaneous BCC in general, whereas reports specifically examining periocular and orbital BCC remain relatively limited. This distinction is important because periocular tumors pose unique therapeutic challenges, and their management carries implications not only for survival but also for vision and quality of life (23).

Several case reports and small case series have suggested that vismodegib can induce significant regression in periocular and orbital BCC, in some instances allowing for globe preservation or even obviating the need for exenteration (24–26). Moreover, there is increasing interest in using vismodegib as a neoadjuvant therapy to reduce tumor burden prior to surgical excision, thereby facilitating less invasive procedures and improving functional outcomes (27). However, despite these promising findings, vismodegib therapy is not without limitations. Adverse events such as muscle cramps, alopecia, dysgeusia, weight loss, and fatigue are common, and while often low grade, they frequently result in treatment discontinuation (21).

Given these considerations, the true role of vismodegib in the treatment of periocular and orbital BCC remains to be clearly defined. While isolated reports and small institutional experiences provide encouraging signals, the evidence is fragmented and often limited by small sample sizes, heterogeneous populations, and variable outcome reporting. To date, no comprehensive systematic review and meta-analysis has been undertaken to evaluate the overall efficacy, safety, and durability of vismodegib in this specific clinical setting. The present systematic review and meta-analysis aim to address this gap by pooling the available evidence on vismodegib in periocular and orbital BCC. Specifically, we sought to evaluate treatment response rates, the incidence of adverse events, recurrence patterns, and the frequency with which vismodegib allowed patients to avoid exenteration.

## 2 Materials and methods

### 2.1 Protocol and registration

This systematic review and meta-analysis was conducted according to the Preferred Reporting Items for Systematic Reviews and Meta-Analyses (PRISMA) guidelines (28).

### 2.2 Eligibility criteria

Studies were eligible for inclusion if they evaluated the use of vismodegib in patients with periocular or orbital basal cell carcinoma, including both locally advanced and recurrent cases, with or without orbital invasion. Vismodegib could be administered as monotherapy or as neoadjuvant treatment prior to surgery. Eligible study designs included prospective and retrospective cohort studies, as well as case series with at least seven patients, as smaller case reports were considered unlikely to provide generalizable outcome data.

Studies were excluded if they were review articles, editorials, commentaries, or conference abstracts lacking sufficient clinical or outcome data. Case reports with fewer than seven patients, studies not reporting outcomes specific to periocular or orbital BCC, and studies in which data could not be separated from other tumor sites were also excluded.

In studies where multiple Hedgehog pathway inhibitors (e.g., vismodegib and sonidegib) were used, only data specific to vismodegib-treated patients were included whenever separable. Studies with non-separable aggregate data were included only if vismodegib represented the predominant treatment modality.

### 2.3 Study selection

**Figure 1:**
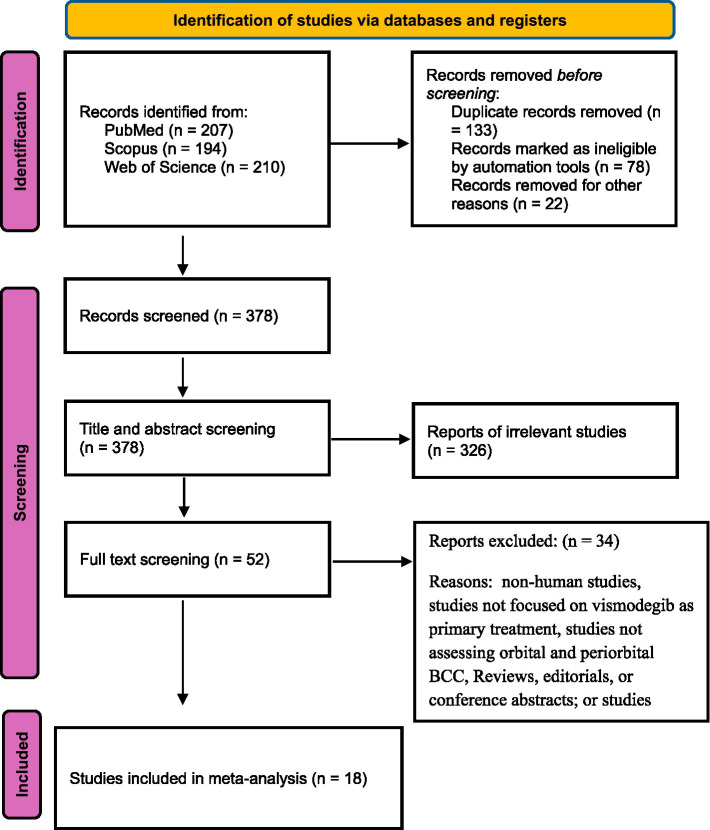
PRISMA flowchart of included studies.

Two independent reviewers screened all titles and abstracts retrieved from the search manually, without the use of automated screening tools or artificial intelligence-assisted methods. Full-text articles of potentially eligible studies were then reviewed in detail against the inclusion and exclusion criteria. Disagreements were resolved by consensus or consultation with a third reviewer. Reasons for exclusion at the full-text stage were documented in the PRISMA flowchart (Figure 1).

### 2.4 Data extraction

Data was extracted independently by two reviewers using a standardized Excel sheet online form. The extracted information included study characteristics (study ID, country, study design, sample size, and age [mean/median ± SD or range]); tumor characteristics (tumor site, tumor size, histological subtype, and primary versus recurrent disease); treatment details (treatment duration and follow-up duration); outcomes (complete response, partial response, recurrence, and exenteration performed); safety outcomes (any adverse event, grade ≥3 adverse event, treatment discontinuation, alopecia, muscle spasm, decreased appetite/anorexia, and weight loss); and other details such as the main findings as reported by the authors. All extracted data were cross-checked for accuracy, and any discrepancies were resolved through discussion.

Where studies included patients treated with different Hedgehog inhibitors, outcome data were extracted preferentially for vismodegib-treated cohorts; if stratified data were unavailable, this was documented and considered during interpretation.

### 2.5 Risk of bias assessment

Given that the included studies were observational in design, methodological quality was evaluated using tools appropriate to each study type. Cohort studies were assessed using the Newcastle–Ottawa Scale (NOS) (29), which evaluates methodological quality across three domains: (1) selection of study participants, including representativeness of the exposed cohort and ascertainment of exposure; (2) comparability of cohorts based on study design or statistical adjustment for potential confounders; and (3) outcome assessment, including adequacy of follow-up duration and completeness of outcome reporting. Each study was assigned a score ranging from 0 to 9 stars, with higher scores indicating lower risk of bias (29).

Case series studies were appraised using the Joanna Briggs Institute (JBI) critical appraisal checklist for case series, which examines key methodological aspects such as clearly defined inclusion criteria, standardized and reliable measurement of the condition, consecutive inclusion of participants, completeness of participant inclusion, adequacy of demographic and clinical reporting, clarity of outcome measurement, and appropriateness of statistical analysis (30).

Two reviewers independently performed all quality assessments, and discrepancies were resolved through discussion or consultation with a third reviewer when necessary. The results of the risk-of-bias evaluation were summarized in Tables 1, 2.

**Table 1:** Methodological quality assessment of cohort studies using the Newcastle–Ottawa Scale (NOS).

Study ID	Selection (max 4)	Comparability (max 2)	Outcome (max 3)	Total (max 9)
Bengoa-Gonza ´lez (12)	☆☆☆	☆	☆☆☆	☆☆☆☆☆☆☆
Demirci (50)	☆ ☆ ☆	☆	☆ ☆	☆ ☆ ☆ ☆ ☆ ☆
Eiger-Moscovich (51)	☆ ☆ ☆	☆	☆ ☆ ☆	☆ ☆ ☆ ☆ ☆ ☆
Giorgi (52)	☆ ☆☆	☆	☆ ☆	☆ ☆ ☆ ☆ ☆
Ishai (41)	☆ ☆ ☆	☆	☆ ☆☆	☆ ☆ ☆ ☆ ☆ ☆☆
Kahana (27)	☆ ☆ ☆	☆	☆ ☆	☆ ☆ ☆ ☆ ☆ ☆
Sagiv (55)	☆ ☆☆	☆	☆ ☆	☆ ☆ ☆ ☆ ☆☆
Sagiv (56)	☆ ☆	☆	☆ ☆	☆ ☆ ☆ ☆ ☆
Tiosano (42)	☆ ☆ ☆ ☆	☆	☆ ☆	☆ ☆ ☆ ☆ ☆ ☆ ☆
Unsworth (57)	☆ ☆ ☆ ☆	☆	☆ ☆	☆ ☆ ☆ ☆ ☆ ☆ ☆
Xavier (60)	☆☆☆	☆	☆☆☆	☆☆☆☆☆☆☆

*Indicates the score assigned using the Newcastle–Ottawa Scale (NOS) for assessing the quality of included observational studies; higher number of stars reflects better methodological quality and lower risk of bias.

**Table 2:** Methodological quality assessment of case series using the Joanna Briggs Institute (JBI) critical appraisal checklist.

Study ID	Were there clear criteria for inclusion in the case series?	Was the condition measured in a standard, reliable way for all participants included in the case series?	Were valid methods used for identification of the condition for all participants included in the case series?	Did the case series have consecutive inclusion of participants?	Did the case series have complete inclusion of participants?	Was there clear reporting of the demographics of the participants in the study?	Was there clear reporting of clinical information of the participants?	Were the outcomes or follow-up results of cases clearly reported?	Was there clear reporting of the presenting site(s)/clinic(s) demographic information?	Was statistical analysis appropriate?	Final score
Curragh (24)	Yes	Yes	Yes	No	Yes	Yes	Yes	Yes	Yes	Yes	**9**
Gill (25)	Yes	Yes	Yes	Yes	Yes	Yes	No	Yes	No	Yes	**8**
González (26)	Yes	Yes	No	Yes	Yes	Yes	Yes	No	Yes	Yes	**8**
Oliphant (53)	Yes	Yes	Yes	No	Yes	Yes	Yes	Yes	Yes	Yes	**9**
Ozgur (54)	Yes	Yes	Yes	Yes	Yes	Yes	No	Yes	No	Yes	**8**
Villani (58)	Yes	Yes	Yes	Yes	Yes	Yes	Yes	Yes	Yes	Yes	**10**
Wong (59)	Yes	Yes	Yes	Yes	Yes	No	Yes	Yes	No	Yes	8

### 2.6 Data synthesis and statistical analysis

A qualitative synthesis was first undertaken to provide a descriptive overview of study and patient characteristics, including demographic features, tumor site and histology, treatment duration, and follow-up. Treatment outcomes and adverse events were tabulated for clarity. For quantitative synthesis, we conducted a single-arm meta-analysis pooling proportions of key clinical outcomes, specifically complete response, partial response, recurrence, and orbital exenteration, as well as safety outcomes including any adverse event, grade ≥3 adverse events, treatment discontinuation, alopecia, muscle spasms, decreased appetite or anorexia, and weight loss.

Meta-analyses were performed using OpenMeta Analyst (Center for Evidence Synthesis in Health, Brown University School of Public Health, Providence, Rhode Island, United States) (31), employing a random-effects model to account for anticipated clinical and methodological heterogeneity between studies. Pooled incidence rates were calculated with corresponding 95% confidence intervals (CI). Between-study heterogeneity was quantified using the I^2^ statistic, with thresholds of 25, 50, and 75% interpreted as low, moderate, and high heterogeneity, respectively. Due to the limited number of included studies and small sample sizes, we did not formally assess publication bias using funnel plots or statistical tests such as Egger’s regression. All analyses were stratified by reported outcomes, and results were presented in forest plots to allow direct comparison across studies. For each outcome, the number of events and total sample size were extracted from individual studies and pooled using a single-arm meta-analytic approach to estimate overall incidence proportions. Forest plots were generated to visually display the effect size for each study, corresponding 95% confidence intervals, and the pooled random-effects estimate. The graphical representation allows comparison of outcome variability across studies and facilitates interpretation of between-study heterogeneity. Results were interpreted in light of study-level methodological differences, variation in follow-up duration, and heterogeneity in patient populations.

## 3 Results

### 3.1 Study selection

The database search initially yielded 611 records. After removal of duplicates, 378 articles were screened by title and abstract, of which 326 were excluded because they did not involve periocular/orbital basal cell carcinoma, were not related to vismodegib, or were reviews/letters without primary data. 52 full-text articles were assessed for eligibility. Of these, 34 were excluded. Finally, 18 studies comprising a total of 764 patients were included in the systematic review and quantitative meta-analysis. A PRISMA flow chart illustrating the selection process is provided in Figure 1.

### 3.2 Study characteristics

The included studies were published between 2013 and 2024 and originated from Europe (Spain, Portugal, Italy, United Kingdom), North America (United States, Canada), South America (Argentina), the Middle East, and Australia. Study designs consisted of 11 cohort studies and 7 case series. Sample sizes ranged from 7 to 244 patients, with a cumulative total of 764 patients. Mean or median ages ranged from 57.6 to 87 years, with most cohorts composed predominantly of elderly patients. Follow-up periods varied between 7.3 and 61 months, with treatment durations typically ranging from 3 to 40 months, depending on disease response and tolerability. All studies administered vismodegib at a daily dose of 150 mg, with some also including sonidegib in small subsets (Table 3).

**Table 3:** Baseline characteristics of included studies evaluating vismodegib in periocular and orbital basal cell carcinoma.

Study ID	Country	Study design	Sample size	Age Mean / median age (± SD / range)	Tumor site	Tumor size	Histological subtype	Primary vs recurrent disease	Treatment duration	Follow-up (months)	outcomes assessed	Main findings
Bengoa-Gonza ´lez 2024 (12)	Spain	Retrospective Cohort	24	72 (42–95)	Periocular with orbital invasion (medial canthus 16/24, lateral canthus 3/24, lower eyelid 4/24, diffuse 1/24)	All T4 per AJCC 8th ed. (orbital invasion; some with bone/paranasal sinus involvement)	Mostly infiltrative (22/24), 1 basosquamous, 1 micronodular	7 primary (29.2%), 17 recurrent (70.8%)	Vismodegib 1–58 months (median adjuvant ~21.5 months); exclusive use 1–12 months; dose 150 mg/day	52 months	Clinical/radiologic response (RECIST), histology, eye preservation, recurrence, survival, treatment-related adverse events, sequelae	Vismodegib achieved CR in monotherapy (1 patient at 8 months); partial responses in others; adjuvant vismodegib prevented recurrence in cases with positive margins; eye preservation possible in many patients; treatment generally well tolerated (muscle cramps, dysgeusia, alopecia most common); one death due to tumor progression, rest had no recurrences; several deaths from unrelated causes.
Curragh 2020 (24)	Australia	Case series	8	57.6 (24–81)	Medial canthus (5), lower eyelid (2), lateral canthus (1)	Pre-treatment simulated excision (tumor + 3 mm margin) mean 27.3 ± 8.6 mm vs. final excised area 19.1 ± 8.5 mm (mean reduction ~8 mm; 2/8 ≤ 3 mm reduction)	Nodular (4), infiltrative (3), mixed superficial/nodular (1)	Primary (6), recurrent (2; both previously surgically treated, no prior RT)	Median 6 months (mean 5.1 ± 1.9; range 3–8) of vismodegib 150 mg/day	13.4 ± 5.2 months	Clinical and histological response, surgical margin status, recurrence, adverse events	All patients had some clinical response (partial in 6/8). Histology showed residual BCC in 6/8, complete regression in 2/8. Clear surgical margins achieved in all; no recurrences during follow-up. Mean predicted excision size was reduced after neoadjuvant vismodegib. Two patients showed squamous differentiation. AEs in 7/8 (87.5%): muscle cramps, alopecia, dysgeusia most common. No exenterations required.
Demirci 2015 (50)	USA	Retrospective Cohort	8	69 (60–86).	Orbital BCC (6 patients, 1 with bone involvement), extensive periocular BCC (2 patients, one with basal cell nevus syndrome)	NR	Basal cell carcinoma: 1 case associated with basal cell nevus syndrome	All recurrent/previously heavily treated; multiple prior surgeries for BCC	Median 8 months (mean 9; range 5–18 months) of vismodegib 150 mg/day	Median 13 months (range 5–21)	Clinical/radiologic response (CR, PR, SD), recurrence, tolerability, adverse events (muscle spasms, alopecia, dysgeusia, dysosmia, GI events)	4/8 achieved complete response (2 periocular, 2 orbital with neoadjuvant/adjuvant vismodegib), 4/8 had partial response (80–95% shrinkage) when vismodegib was used as sole therapy; no recurrences at 19–21 months in neo−/adjuvant patients; periocular cases remained disease-free after 12–18 months; most common side effects were muscle spasms (75%), alopecia (50%), dysgeusia (25%), dysosmia (25%), and GI symptoms; one patient discontinued due to toxicity, one died of unrelated cardiac event.
Eiger-Moscovich 2019 (51)	Israel	Retrospective Cohort	21	76 (59–91)	Orbital BCC (15 patients), periocular BCC (6 patients)	NR	Mostly basal cell carcinoma; 1 basosquamous transformation	All locally advanced; many had multiple prior surgeries, radiotherapy, or exenteration (recurrent/advanced); no distant metastases	Median 9 months (range 1–53 months); CR median 11.5 months, PR median 7.5 months	Median 26 months (range 9–60); mean 17 months after treatment cessation	RECIST 1.1 response (CR, PR, SD), recurrence, need for additional therapy (surgery/radiotherapy/5-FU), adverse events (CTCAE v5.0), survival	20/21 responded (10 CR, 10 PR, 1 SD, no PD); 5 CR patients remained disease-free after treatment cessation (16-month mean follow-up), 3 relapsed at ~8 months; alternating vismodegib + Efudix effective for recurrences; most AEs grade 1–2 (muscle spasm 76%, dysgeusia 57%, alopecia 47%, weight loss 47%); 2 patients developed grade 3–4 hepatotoxicity; 1 death possibly treatment-related (sepsis)
Gill 2013 (25)	Canada	Case series	7	71 (43–100)	Periocular BCC (all infiltrative; 4/7 [57%] with orbital extension)	Mean 3.4 cm (range 1.0–6.0 cm)	Infiltrative basal cell carcinoma (all biopsy-proven)	100% recurrent cases,	Mean 11 weeks (range 4–16 weeks)	Mean 7.3 months (range 5–10 months)	Lesion size reduction (clinical and imaging), ulceration resolution, treatment tolerability, new skin cancers	2 patients (29%) had complete regression, 2 (29%) had >80% partial regression, 2 (29%) had <35% partial regression, and 1 (14%) progressed. Ulceration resolved in 5/7 patients. Of the 4 with orbital extension, 3 showed partial response with tumor shrinkage on imaging. Adverse events in 6/7 patients (alopecia, dysgeusia, cramps, anorexia). Two patients developed new squamous cell carcinomas at uninvolved sites. No disease-related or treatment-related deaths.
Giorgi 2021 (52)	Italy	Retrospective Cohort	15	87 (63–94)	Periocular (medial canthus 8/15, lateral canthus 6/15, lower eyelid 1/15); orbital involvement in 5/15	NR	NR	Mixed – 8 previously treated with surgery only, 2 with surgery + radiotherapy, 5 not eligible for surgery/radiotherapy	Vismodegib 150 mg/day or sonidegib 200 mg/day; median ~12 months for CR; many discontinued after ~5 cycles due to AEs; some on modified/pulse regimens	31 months	Clinical response (CR, PR, SD), orbital involvement regression, eye preservation, recurrence, adverse events	All patients benefited (CR/PR/SD); 3 achieved CR (2 recurred later, 1 remained disease-free); most others had PR; discontinuation common (60%) due to AEs (mainly dysgeusia, muscle spasms, weight loss, fatigue); pulse/alternative dosing-maintained response with improved tolerability; eye-sparing possible; sonidegib well tolerated in 2 patients with good response.
González 2018 (26)	Argentina	Case series	8	76 (60–90)	Medial canthus (62.5%), lower eyelid (25%), lower eyelid + medial canthus (12.5%); 37.5% with conjunctival invasion	Mean 18 mm (range 12–30 mm)	Nodular (62.5%), infiltrative (25%), micronodular (12.5%)	5 primary (62.5%), 3 recurrent (37.5%; 2 after incomplete excision, 1 after MMS)	Vismodegib 150 mg daily until maximal clinical response; mean 4.8 months to maximal response; mean 7.3 months to surgery	Mean 14.4 months (range 8–26)	Clinical response, histologic response after MMS, disease-free survival, adverse events	87.5% achieved clinical complete response; 83.3% of surgical patients had histologic complete response; all patients disease-free at last follow-up; 1 progression requiring exenteration; universal adverse events (100% dysgeusia, 100% muscle spasms, 75% weight loss, 50% alopecia); one withdrawal due to toxicity.
Ishai 2020 (41)	Israel	Retrospective Cohort	244	72.0 (60.0–82.0)	Periocular locally advanced BCC (238/244) and periocular metastatic BCC (6/244)	NR	Locally advanced BCC (97.5%), metastatic BCC (2.5%); Gorlin syndrome in 34 patients (14.3%)	Mixed (not systematically reported; 26.2% had prior radiotherapy; many had multiple prior treatments)	Median exposure 40 weeks (IQR 20–78); 39.7 weeks for POLA-BCC; 92.4 weeks for mBCC	Median 39 weeks (IQR 23.5–56.3)	Response per RECIST (CR, PR, SD, PD), progression-free survival, overall survival, duration of response, safety/adverse events	ORR 67.2% (CR 28.7%, PR 38.5%). No CR among metastatic periocular cases (only PR in 4/6). Adverse events in 95.1% (most common: alopecia 68%, muscle spasms 67.6%, dysgeusia 54.5%, weight loss 49.2%). 23.8% discontinued due to AEs; 9% died during study (mainly unrelated causes). Vismodegib was generally effective and tolerable in POLA-BCC.
Kahana 2021 (27)	USA	Cohort	34	67.1+/−12.2	Medial canthus (22), lateral canthus (3), lower eyelid (8), brow/orbit junction (2)	21.5 mm (range 10–60)	NR	primary	Median 261 days; up to 12 months of oral vismodegib 150 mg daily	12 months	Visual Assessment Weighted Score (VAWS), globe preservation, tumor regression (RECIST v1.1), MRI/CT imaging, histopathology after surgery, adverse events	100% maintained VAWS >21 (globe preservation); 56% achieved complete regression by physical exam, 47% by MRI/CT; histology after surgery showed 67% complete clearance, 22% residual disease with clear margins, 11% residual disease to margins; vismodegib preserved globe and visual function; two recurrences noted at 2-year follow-up.
Oliphant 2020 (53)	UK	Case series	13	75 (43–91)	Periocular BCC — medial canthus (7), lower eyelid (3), both upper & lower eyelids (1), forehead to superior orbit (1), temple to lateral canthus (1); orbital involvement in 7/13 (58%)	NR	Infiltrative (4), nodular (3), nodular/infiltrative (1), micronodular/infiltrative (1), baso-squamous (1), superficial (1), cystic (1), NA (1)	Primary (5/13, 38%), recurrent after prior treatment (8/13, 62%)	Median 7 months (range 2–36), vismodegib 150 mg/day	Median 24 months (range 12–48; mean 30)	Clinical response (CR, PR, progression), recurrence, surgical outcomes (globe-sparing vs. exenteration), adverse events	CR in 5/13 (38%), PR in 8/13 (62%). Three patients (23%) developed recurrence. Six underwent post-vismodegib surgery, three globe-sparing. Three ultimately underwent exenteration. AEs in 11/13 (85%): most common fatigue (46%), dysgeusia, weight loss, reduced appetite, muscle cramps, alopecia. Authors conclude vismodegib is well tolerated, may enable globe-sparing surgery, but recurrence and exenteration risk remain.
Ozgur 2015 (54)	USA	Case series	13	64.5 (33–86)	Periocular/orbital BCC (upper lid, lower lid, medial/lateral canthus, brow; some with orbital extension); also included 2 basal cell nevus syndromes with periocular lesions	Range 1.5 × 1.8 cm to 11.5 × 8 cm (median not reported; detailed sizes in Table 2)	NR	Locally advanced (10 cases, 4 also metastatic) and 2 basal cell nevus syndromes; all not amenable to curative surgery/radiotherapy (recurrent/advanced)	11.5 months (range 7–49 months)	12.5 months (range 7–53 months)	RECIST response (CR, PR, SD, PD), avoidance of orbital exenteration, recurrence/progression, drug-related adverse events (CTCAE v4.0), survival	Overall response rate 86% in locally advanced nonmetastatic BCC, 100% in basal cell nevus syndrome, 33% in metastatic BCC; 3/12 achieved CR (up to 38 months), 6 PR, 3 SD; 5 patients with T3bN0M0 avoided exenteration; 2 progressed after initial response; common AEs included muscle spasms (100%), weight loss (83%), dysgeusia (75%), alopecia (75%); 42% had grade II AEs; 1 patient died of disease progression after 16 months of SD.
Sagiv 2019 (55)	USA	Retrospective Cohort	8	69 (55–84)	Periocular (all T4, invading ocular/orbital/facial structures)	NR	1 nodular, rest combined nodular + infiltrative; 5 with focal squamous differentiation; 2 with perineural invasion	2 primary (25%), 6 recurrent (75%)	14 months (range 4–36) vismodegib (150 mg/day) until surgery	18 months	Clinical response (RECIST v1.1), histologic response, eye preservation, surgical margins, recurrence, adverse events	All achieved eye-sparing surgery with negative margins; 5/8 complete histologic response, 3/8 partial response; no recurrences/metastases at last follow-up; treatment generally well tolerated with mostly grade 1–2 AEs, 2 patients discontinued early due to side effects but still had successful eye-preserving surgery.
Sagiv 2019 (56)	USA	Retrospective Cohort	42	63 ± 11	Locally advanced periocular BCC (all T4 per AJCC 8th ed.; orbital extension common)	NR	NR	23/42 (55%) presented with recurrent tumors (69% before vs. 48% after FDA approval, *p* = 0.317)	Vismodegib median ~8–13 months (mean 8 ± 5 before vs. 13 ± 3 months after approval; *p* = 0.605)	Median 61 months (range 4–108) before approval vs. 12 months (1–48) after approval	Exenteration rates, eye preservation, recurrence, survival status, vismodegib use, radiation use	Orbital exenteration is significantly less common after vismodegib approval (46% before vs. 10% after; *p* = 0.016); vismodegib use significantly more common after approval (69% vs. 23%; *p* = 0.008); trend toward greater eye preservation after approval (83% vs. 54%, *p* = 0.066); recurrence rates similar (15% before vs. 7% after; *p* = 0.576); no significant difference in survival outcomes.
Tiosano 2022 (42)	Israel	Retrospective Cohort	244	67.00 [52.50, 82.00]	Periocular locally advanced BCC (POLA BCC); 258 LA-BCC, 6 metastatic BCC	Grouped: ≤10 mm (8.3%), >10–20 mm (33.7%), >20–30 mm (25%), >30 mm (39%)	Locally advanced BCC (96.7%); small proportion metastatic (2.3%)	Mixed (not clearly reported; many had prior radiotherapy)	Variable: data analyzed at 3 and 6 months (follow-up extended up to several years)	Longitudinal; main predictive cutoffs at 3 months and 6 months, extended analyses across first year	Tumor size reduction (RECIST v1.1), complete response (CR), partial response (PR), progressive disease (PD), stable disease (SD); predictive modeling for CR	92.4% of tumors reduced in size; smaller tumors (≤20 mm) had higher CR rates (54.5% group 1; 39.6% group 2) vs. larger tumors (>30 mm, 22.6%). PD observed only in largest tumors (>30 mm). ROC analysis: ≥20% reduction at 3 months → 82.8% probability of CR; ≥67.7% reduction at 6 months → 95.4% probability of CR. Prior radiotherapy reduced likelihood of CR.
Unsworth 2022 (57)	USA	Cohort	34	67.1+/−12.2	Medial canthus (63%), lateral canthus (8.5%), lower eyelid (23%), brow/orbit (5.5%)	21.5 mm (range 10–60)	NR	primary	Vismodegib until surgery	24 months	Clinical response, histologic response, eye preservation, recurrence, molecular markers (Gli1, SMO/PTCH1 mutations)	56% clinical CR; 67% histological CR; keratin-positive Gli1 + micro-lesions detected even in CR; resistant SMO mutations (e.g., W535L) identified; one recurrence at 2 years due to resistant clone expansion.
Villani 2020 (58)	Italy	Case series	13	76.5 ± 13.7	Periocular laBCC — lateral canthus (5), lower eyelid (4), both eyelids (2), forehead extending to orbit (2); orbital involvement in 3 cases	NR	Ulcerated (8/13), nodular (3/13), morpheiform (2/13)	9/13 (69.2%) had previous surgery; 2/13 (15.3%) prior radiotherapy; 2/13 (15.3%) topical treatments → majority recurrent	Median 7 months (range 1–21 months), vismodegib 150 mg/day	12 months	Clinical response (CR, PR, SD, PD), recurrence, adverse events, role as neoadjuvant	CR in 7/13 (53.8%), PR in 4/13 (30.8%), no response (SD/PD) in 2/13 (15.4%); ORR (CR + PR) = 84.6%. Recurrence occurred in 4 patients (30.8%) within 1 year. AEs: muscle pain (92.3%), dysgeusia (84.6%), alopecia (30.7%). 4/13 underwent surgery post-vismodegib. Authors conclude vismodegib is effective, safe, and can serve as neoadjuvant therapy in periocular/orbital laBCC.
Wong 2017 (59)	UK	Case series	15	74 (44–90)	Periocular and orbital (67% with orbital involvement)	Mean longest dimension 51 mm (range 8–115 mm)	NR	8 primary (53%), 7 recurrent (47%; prior surgery and/or radiotherapy, including 1 basal-cell nevus syndrome)	Mean 13 months (range 2–40 months) of vismodegib 150 mg daily	Mean 36 months (range 14–52)	Clinical response, progression-free survival, disease-free survival, orbital salvage, adverse events	67% complete response, 20% partial response, 13% progressive disease; 1 recurrence after CR; 3 patients required orbital exenteration (all with orbital invasion); partial response enabled curative resection in 1 case; most adverse events were mild/moderate (93% with dysgeusia, muscle spasm, alopecia, asthenia); treatment discontinued in 73% mainly due to AEs or progression.
Xavier 2021 (60)	Portugal	Retrospective Cohort	13	73 (54–96)	Periocular	NR	NR	NR	Median 10.5 months (range 2.7–41.2) of vismodegib 150 mg/day	Median 15.9 (range 2.7–53.5)	Objective clinical response (CR, PR, SD, PD), duration of response, progression, death, treatment tolerability	ORR 76.9% (CR 30.8%, PR 46.2%); SD 23.1%. Median duration of response 13 months (range 2.6–52.5). PD in 38.5% (5/13), median 19 months after starting therapy. Toxicity in 84.6% (muscle spasms 69%, fatigue 61.5%, alopecia 46.2%, dysgeusia 46.2%); 61.5% required temporary or permanent treatment interruptions, 1 patient discontinued permanently. Four deaths occurred during follow-up, only 1 attributable to BCC progression.

AJCC, American Joint Committee on Cancer; AEs, Adverse Events; BCC, Basal Cell Carcinoma; CR, Complete Response; CT, Computed Tomography; CTCAE, Common Terminology Criteria for Adverse Events; FU, Follow-Up; GI, Gastrointestinal; LA-BCC, Locally Advanced Basal Cell Carcinoma; MMS, Mohs Micrographic Surgery; MRI, Magnetic Resonance Imaging; mBCC, Metastatic Basal Cell Carcinoma; NR, Not Reported; ORR, Overall Response Rate; PD, Progressive Disease; POLA-BCC, Periocular Locally Advanced Basal Cell Carcinoma; PR, Partial Response; RECIST, Response Evaluation Criteria in Solid Tumors; RT, Radiotherapy; SD, Stable Disease; VAWS, Visual Assessment Weighted Score.

### 3.3 Tumor characteristics

Tumors were primarily located in the medial canthus (most common site across studies), followed by the lower eyelid and, less frequently, the lateral canthus or upper eyelid. Orbital invasion was present in a substantial proportion of patients, particularly in the larger cohorts. Reported tumor sizes ranged from 12 mm to over 115 mm, with many categorized as T4 disease under AJCC 8th edition due to orbital and bony involvement. Histological subtypes included infiltrative, nodular, micronodular, mixed patterns, and occasional basosquamous transformations. Both primary tumors and recurrent tumors were represented; recurrence after prior surgery or radiotherapy was particularly frequent, reaching 70.8% in Bengoa-González 2024 and 75% in Sagiv 2019 (Table 3).

### 3.4 Risk of bias assessment

Risk of bias assessment was performed using the Newcastle–Ottawa Scale (NOS) for cohort studies and the Joanna Briggs Institute (JBI) checklist for case series. All cohort studies were rated as high quality (7–9 stars) except for two studies, which were graded as moderate quality (4–6 stars) due to incomplete follow-up and lack of clarity regarding comparability of cohorts (Table 1). All case series achieved a high-quality rating according to JBI criteria, with clearly defined inclusion criteria, valid outcome measurement, and appropriate clinical detail. Overall, the methodological quality was deemed acceptable (Table 2).

### 3.5 Data analysis

- Complete Response

**Figure 2:**
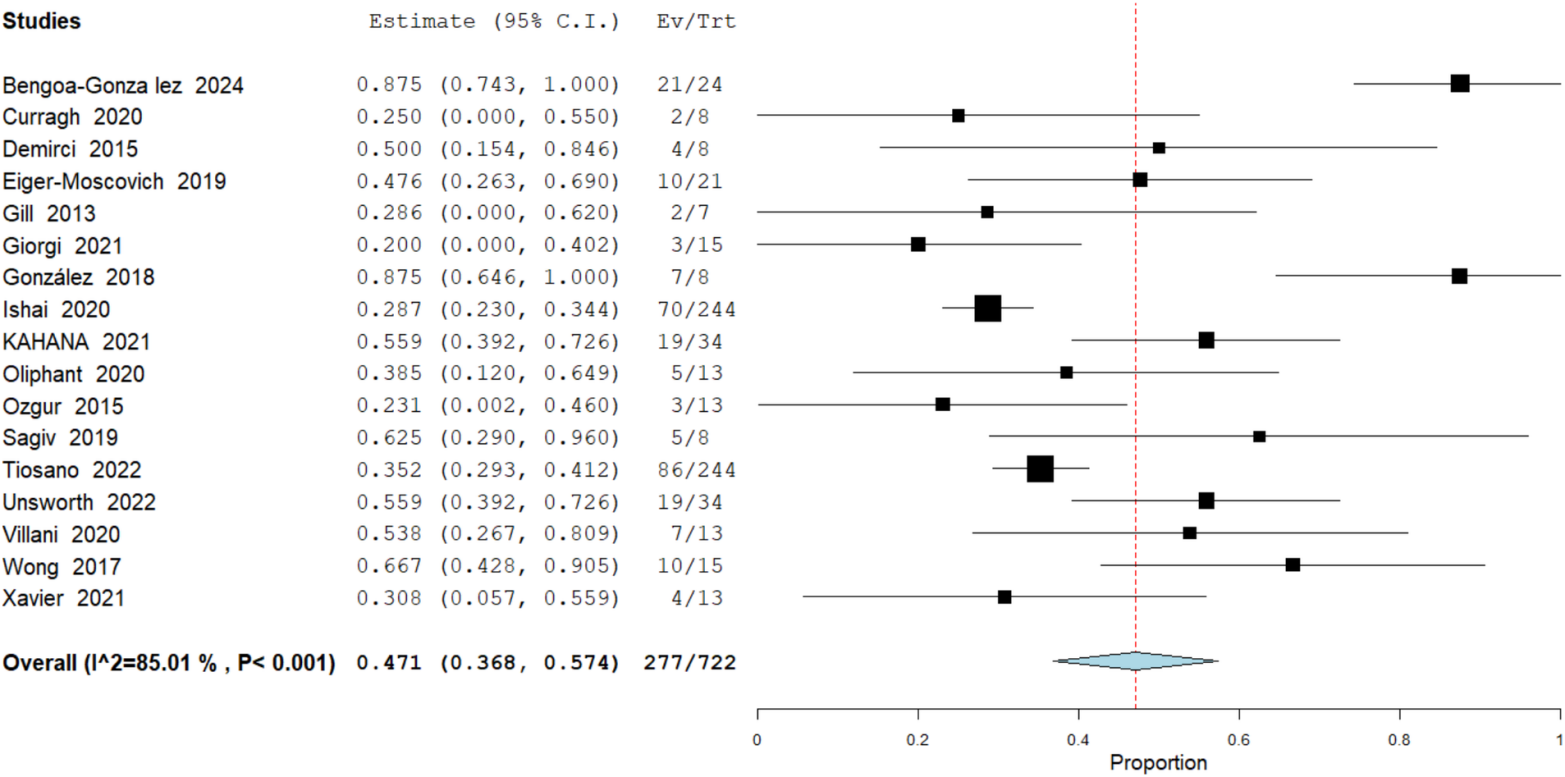
Meta analysis of incidence of CR in patients treated with vismodegib.

The pooled incidence of complete response (CR) from 722 patients across 17 studies to vismodegib among patients with periocular and orbital BCC was 38.36% (95% CI: 36.6–57.4). Across individual cohorts. Heterogeneity was high (I^2^ = 85.01%), indicating some variability in response across studies. These findings suggest vismodegib can achieve tumor clearance in a substantial proportion of patients (Figure 2).

- Partial Response

**Figure 3:**
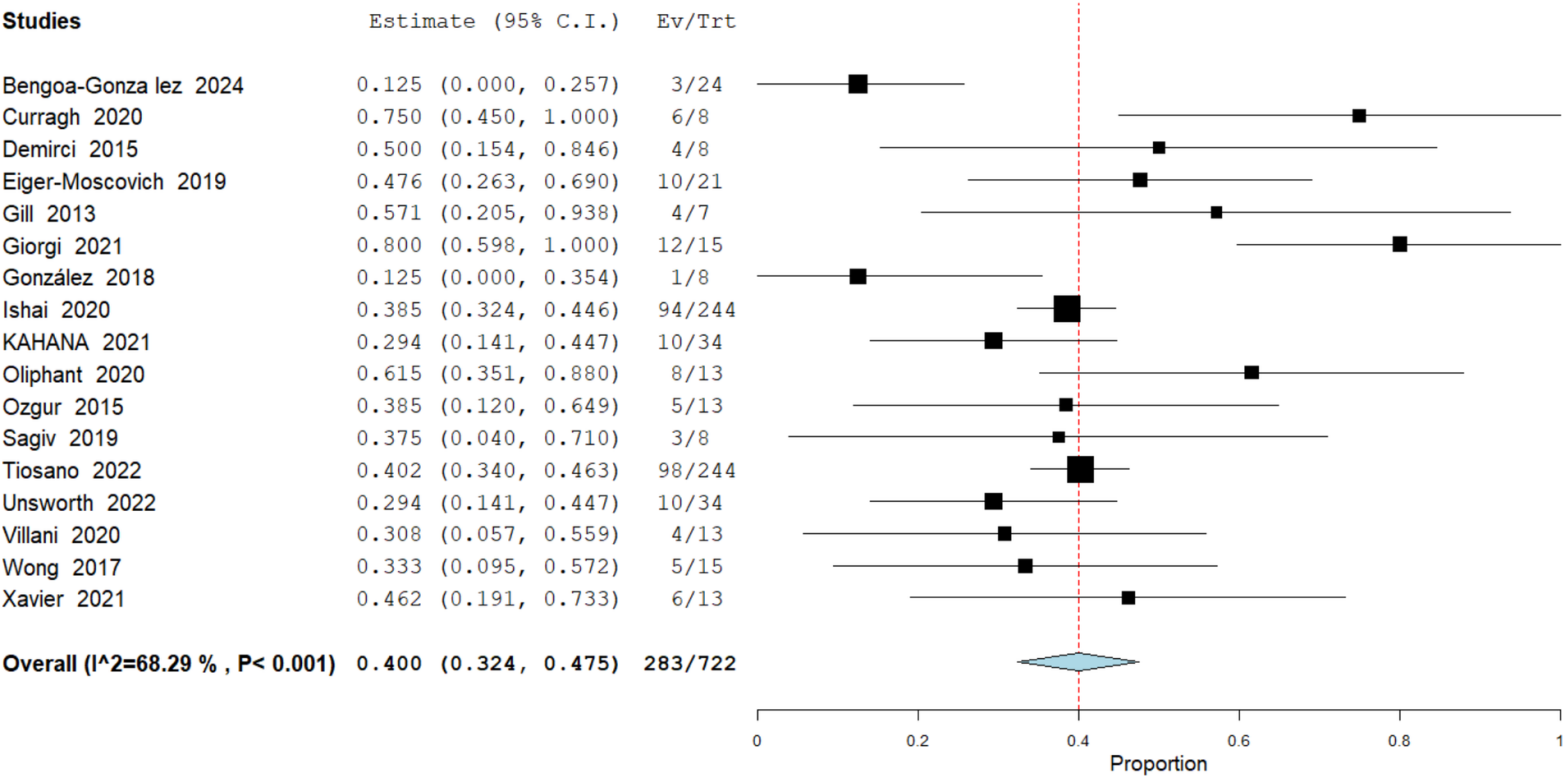
Meta analysis of incidence of PR in patients treated with vismodegib.

Data analysis from 722 patients across 17 studies of partial response (PR) was observed in a pooled 39.19% (95% CI: 32.4–47.5) of cases. Several patients achieving PR subsequently underwent conservative surgical excision with histologically clear margins, highlighting the neoadjuvant potential of vismodegib. Heterogeneity was moderate (I^2^ = 68.29%), suggesting consistent reporting across cohorts (Figure 3).

- Exenteration Performed

**Figure 4:**
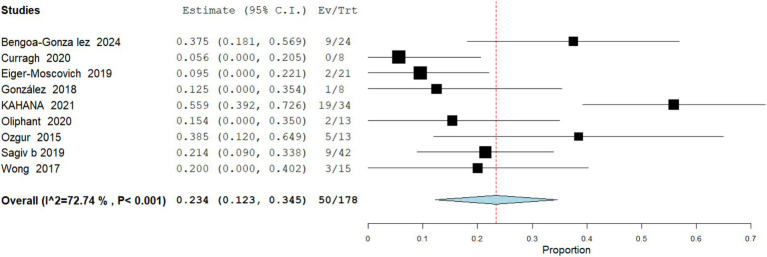
Meta analysis of incidence of exenteration performed in patients treated with vismodegib.

Data analysis from 178 patients across 9 studies demonstrate that the pooled rate of orbital exenteration following vismodegib treatment was 28.08% (95% CI: 12.3–34.5). Heterogeneity for this outcome was moderate (I^2^ = 72.7%), reflecting variability across studies (Figure 4).

- Recurrence

**Figure 5:**
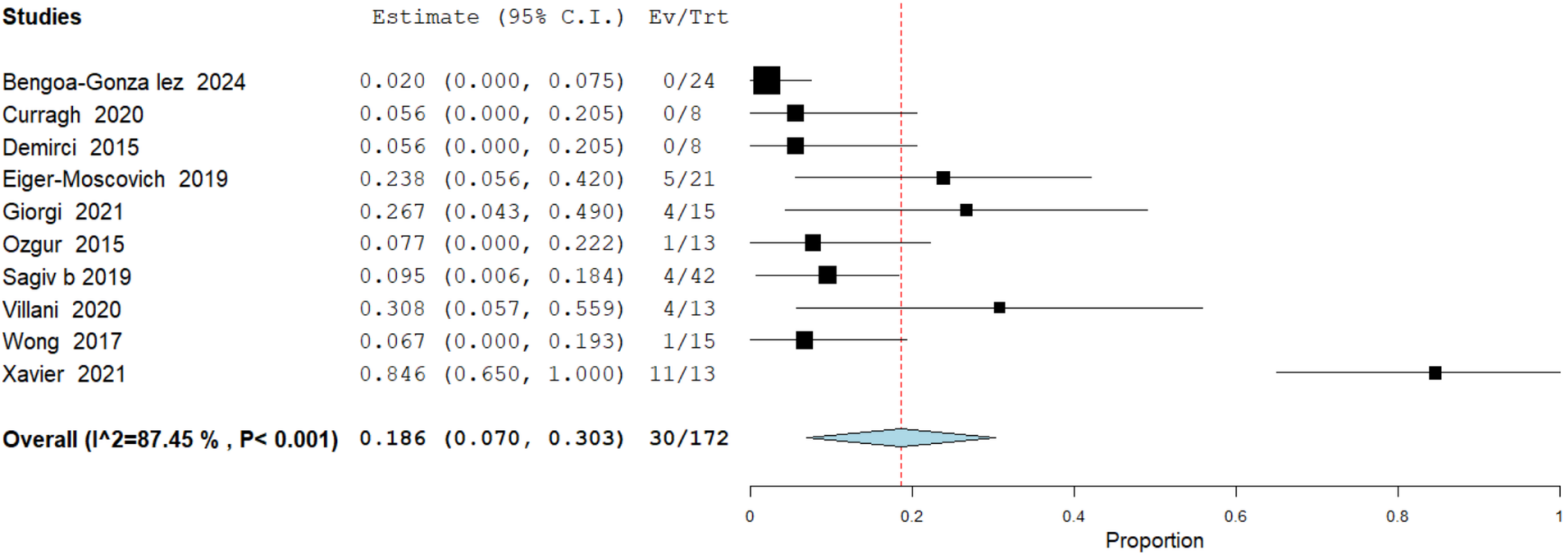
Meta analysis of incidence of recurrence in patients treated with vismodegib.

Data analysis from 172 patients across 10 studies demonstrate that tumor recurrence after vismodegib discontinuation occurred in 17.44% (95% CI: 7–30.3) of patients, most commonly within 12–24 months. Recurrence was more frequent in patients with PR compared to those who achieved CR. Some relapsed patients responded to retreatment, while others required surgical intervention. Heterogeneity was high (I^2^ = 87%; Figure 5).

- Any Adverse Event

**Figure 6:**
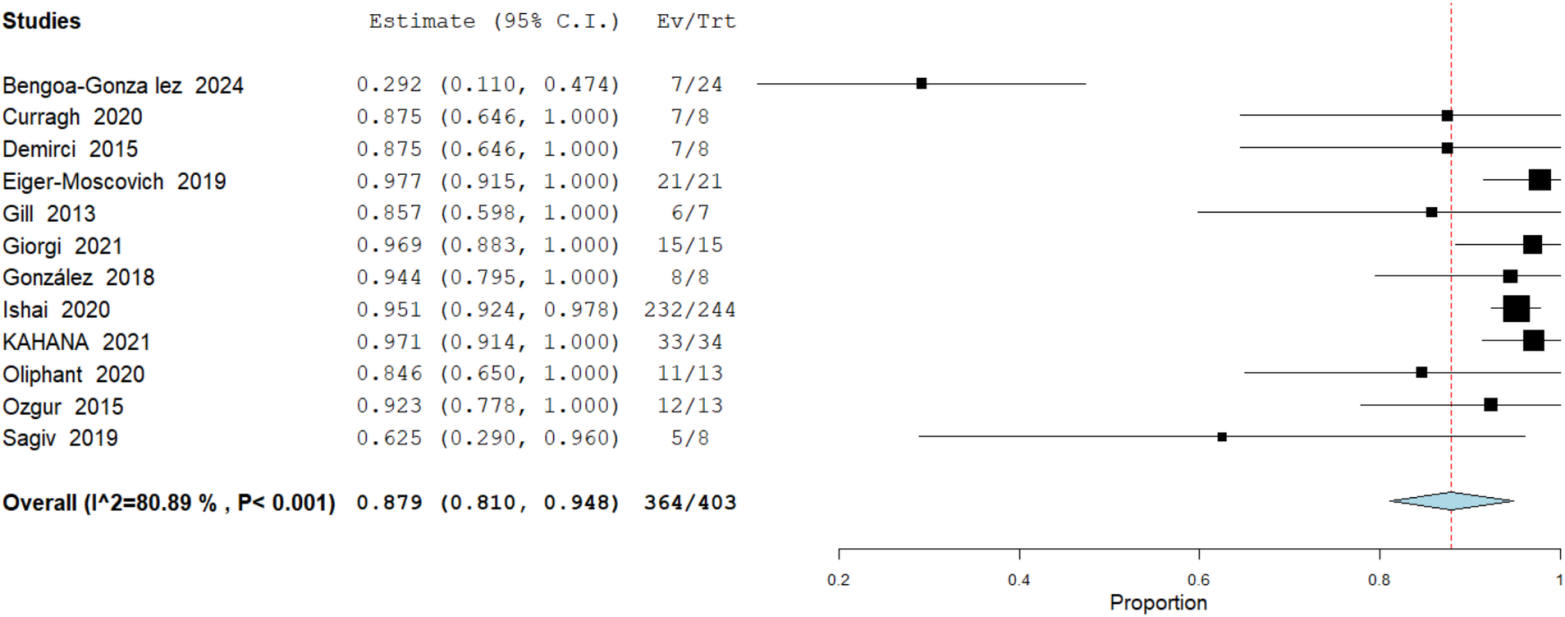
Meta analysis of incidence of any adverse event in patients treated with vismodegib.

Data analysis from 403 patients across 12 studies demonstrate that nearly all patients experienced at least one treatment-related adverse event, with a pooled incidence of 90.32% (95% CI: 81–94.8). The most common were alopecia, muscle spasms, dysgeusia, weight loss, and anorexia. Most AEs were grade 1–2. Heterogeneity was high (I^2^ = 80.8%; Figure 6).

- Grade ≥3 Adverse Events

**Figure 7:**
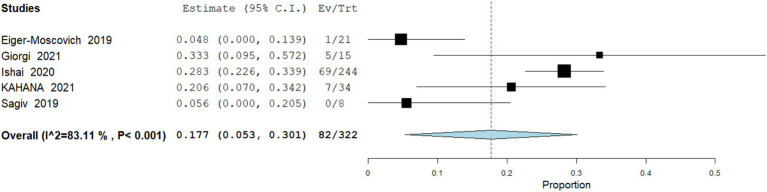
Meta analysis of incidence of grade ≥3 adverse events in patients treated with vismodegib.

Data analysis from 322 patients across 5 studies demonstrate that severe adverse events (grade ≥3) were reported in 25.46% (95% CI: 5.3–30.1) of patients, including significant weight loss, disabling spasms, and profound fatigue. Heterogeneity was high (I^2^ = 83.1%; Figure 7).

- Treatment Discontinuation

**Figure 8:**
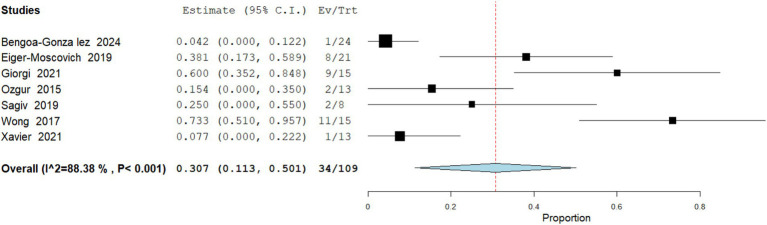
Meta analysis of incidence of treatment discontinuation in patients treated with vismodegib.

Data analysis from 109 patients across 7 studies demonstrate that treatment discontinuation occurred in 31.2% (95% CI: 11.3–50.1) of patients, most often due to cumulative toxicity rather than disease progression. Heterogeneity was high (I^2^ = 88.3%; Figure 8).

- Alopecia

**Figure 9:**
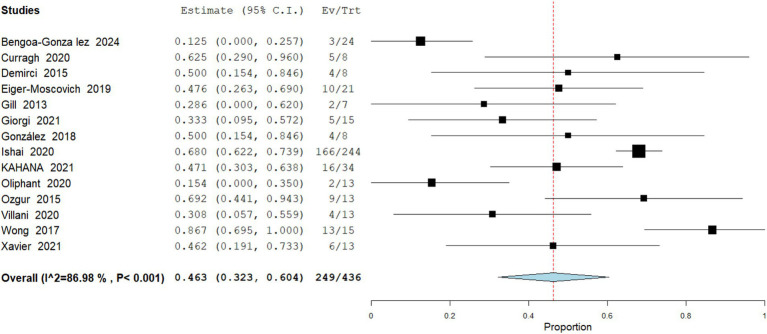
Meta analysis of incidence of alopecia in patients treated with vismodegib.

Data analysis from 436 patients across 14 studies demonstrate that alopecia was reported in 57.11% (95% CI: 32.3–60.4) of patients. Although not life-threatening, it contributed significantly to reduced quality of life and patient-driven treatment discontinuation. Heterogeneity was high (I^2^ = 86.9%; Figure 9).

- Muscle Spasms

**Figure 10:**
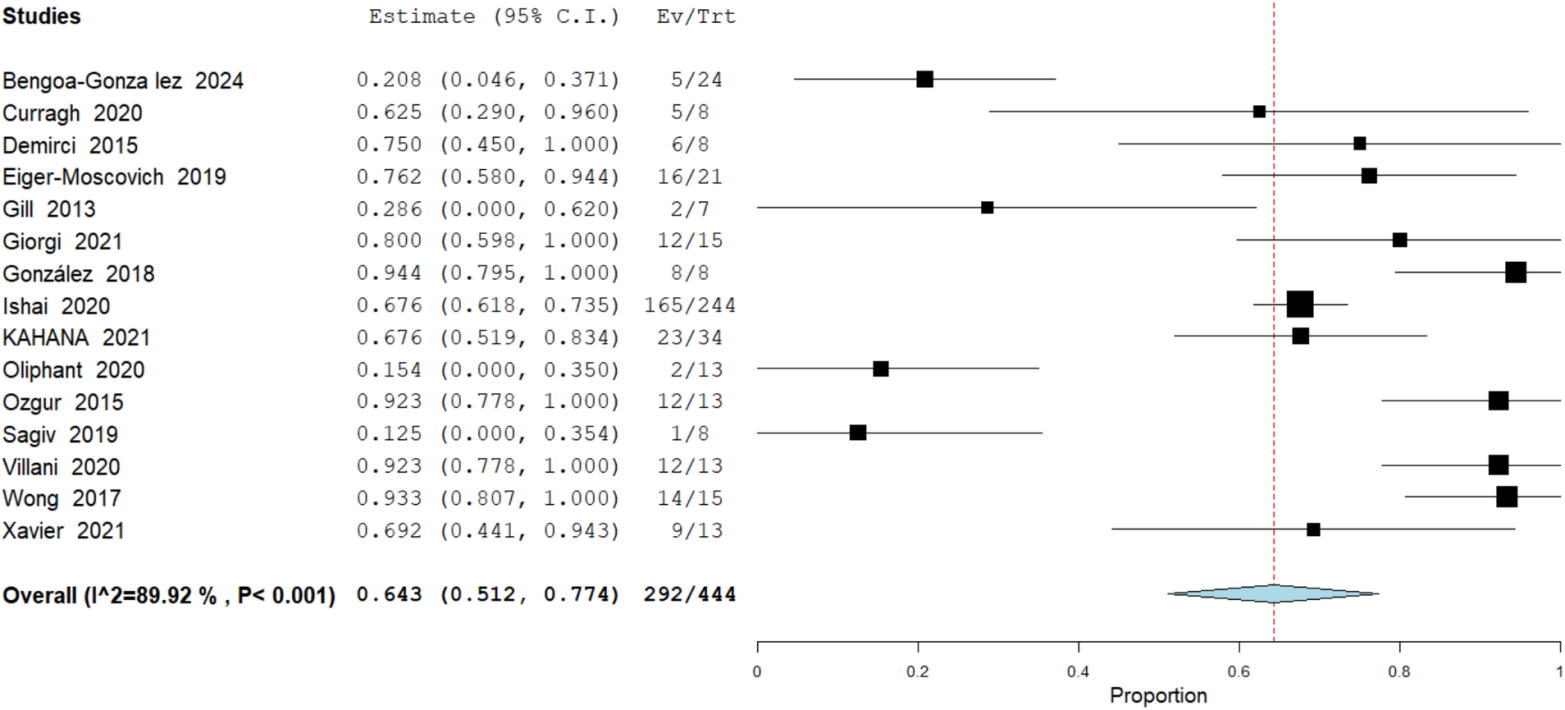
Meta analysis of incidence of muscle spasms in patients treated with vismodegib.

Data analysis from 444 patients across 15 studies demonstrate that Muscle spasms were among the most frequent toxicities, with a pooled rate of 65.8% (95% CI: 51.2–77.4). They were generally low-grade but often persistent, leading to discomfort and drug interruption in some patients. Heterogeneity was high (I^2^ = 89.9%; Figure 10).

- Decreased Appetite / Anorexia

**Figure 11:**
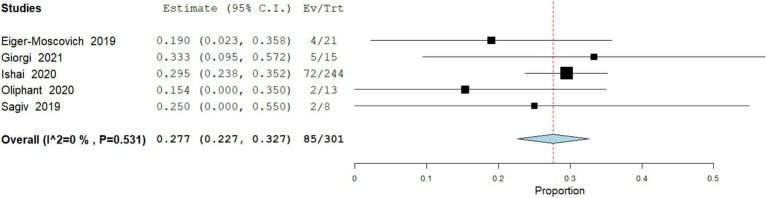
Meta analysis of incidence of decreased appetite / anorexia in patients treated with vismodegib.

Data analysis from 301 patients across 5 studies demonstrate that Appetite loss or anorexia occurred in 28.2% (95% CI: 22.7–32.7) of patients. There was no heterogeneity (I^2^ = 0%; Figure 11).

- Weight Loss

**Figure 12:**
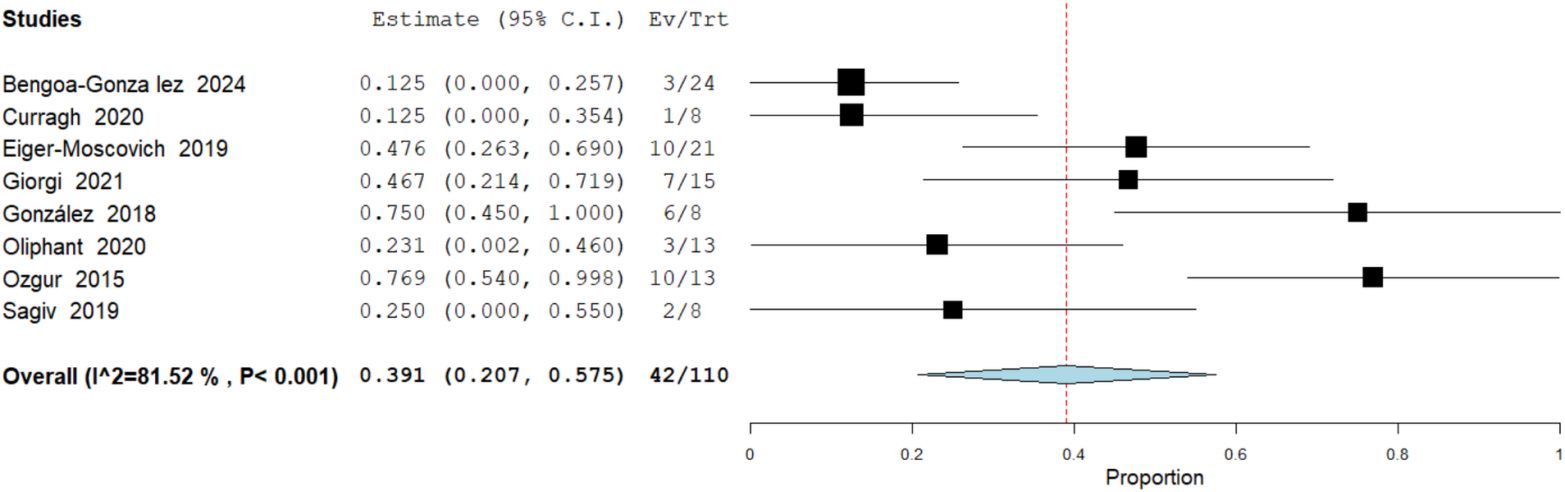
Meta analysis of incidence of weight loss in patients treated with vismodegib.

Data analysis from 110 patients across 8 studies demonstrate that Weight loss was noted in a pooled 38.2% (95% CI: 20.7–57.5) of patients and was frequently linked to anorexia, dysgeusia, or chronic spasms. Severe cases contributed to the grade ≥3 AE category. Heterogeneity was high (I^2^ = 81.5%; Figure 12).

## 4 Discussion

This systematic review and meta-analysis of 18 studies encompassing 764 patients provides the most comprehensive synthesis to date of vismodegib use in periocular and orbital BCC. Our pooled analysis demonstrated that vismodegib achieves meaningful tumor regression, with a CR in 38.4% of patients and PR in 39.2%. Importantly, many patients with PR subsequently underwent less invasive surgery with clear margins, underscoring the drug’s neoadjuvant potential. Despite these encouraging results, recurrence following treatment discontinuation remained a concern, occurring in 17.4% of patients, most often within 12–24 months. While vismodegib facilitated globe preservation in a subset of patients, exenteration was still required in 28.1%, reflecting the aggressive nature of periocular tumors in some cases. Toxicity was nearly universal, with 90% of patients experiencing at least one adverse event. The most frequent were muscle spasms (65.8%) and alopecia (57.1%), while severe grade ≥3 events occurred in 25.5% of cases. Treatment discontinuation due to adverse effects was common, affecting nearly one-third of patients (31.2%).

The primary objective of the present study was to provide a comprehensive synthesis of the currently available clinical evidence regarding the efficacy and safety of vismodegib in periocular and orbital basal cell carcinoma. While this analysis consolidates the best available observational data to inform present-day clinical decision-making, it also highlights several areas requiring further investigation. Future research should focus on prospective multicenter registries with standardized reporting of tumor stage, response criteria, treatment duration, and long-term outcomes. Comparative effectiveness studies evaluating vismodegib against alternative Hedgehog inhibitors or multimodal treatment approaches may help define optimal sequencing strategies. In addition, translational studies exploring molecular predictors of response and resistance could facilitate more individualized treatment selection and improve long-term disease control.

A key consideration in interpreting our findings is the substantial heterogeneity observed across several pooled outcomes. This variability is likely multifactorial and reflects important differences in study populations and treatment contexts. First, tumor characteristics varied considerably, with included cohorts comprising both primary and recurrent disease, as well as differing degrees of orbital invasion and tumor size, which are known to influence treatment response and prognosis. Second, prior treatments, particularly radiotherapy and multiple surgical interventions, may have altered tumor biology and responsiveness to Hedgehog pathway inhibition. Third, a small subset of studies included patients with metastatic disease, which may have further contributed to variability in outcomes. In addition, heterogeneity in treatment duration, dosing modifications, and follow-up periods may have influenced both response rates and the detection of recurrence. These factors collectively limit direct comparability between studies and likely account for the high I^2^ values observed in several analyses.

The clinical implications of our findings are particularly relevant for the management of periocular and orbital BCC, where standard surgical excision may lead to profound functional and cosmetic morbidity (32). Vismodegib has emerged as a critical therapeutic option in this context, offering the possibility of tumor regression without resorting to orbital exenteration (33). Our pooled data demonstrate that nearly four in 10 patients achieve complete remission and an additional four in 10 achieve partial regression, translating into a significant opportunity to downstage advanced tumors. This neoadjuvant role of vismodegib is of special importance, as it allows surgeons to perform more conservative resections with histologically negative margins in cases that would otherwise necessitate disfiguring procedures (34). Nevertheless, the persistence of a 28% exenteration rate underscores that vismodegib is not curative for all patients and should be positioned as a component of a multimodal strategy rather than a standalone replacement for surgery. The high rate of adverse events, particularly alopecia, muscle spasms, and weight loss, further emphasizes the importance of patient counseling and expectation management before treatment initiation. For elderly patients with frailty or significant comorbidities, clinicians must carefully balance the potential for visual organ preservation with the impact of chronic drug-related toxicity on overall quality of life (27). The observed recurrence rate of 17% following treatment discontinuation highlights the need for long-term follow-up and raises questions about the optimal duration of therapy, as continuous exposure is often limited by tolerability.

The development of vismodegib represents a paradigm shift in the management of advanced BCC (18, 19). BCC is driven by aberrant activation of the Hedgehog signaling pathway, a molecular cascade that regulates embryonic development but is normally dormant in adult tissues (16). Mutations in the *PTCH1* or *SMO* genes lead to constitutive pathway activation, driving uncontrolled tumor growth (17). Vismodegib, a first-in-class oral SMO, was designed to block this aberrant signaling at its source (20). Following promising results in early-phase trials, vismodegib became the first Hedgehog pathway inhibitor approved by the US Food and Drug Administration (FDA) in 2012 for the treatment of metastatic or locally advanced BCC in patients unsuitable for surgery or radiotherapy (21). Its use was quickly extended to periocular and orbital disease, where conventional surgical management often entails orbital exenteration, a procedure associated with devastating functional and psychosocial consequences. By offering a non-surgical route to tumor regression, vismodegib provides both a therapeutic alternative and a neoadjuvant bridge to less mutilating surgery (35).

In addition to vismodegib, sonidegib is another orally administered Smoothened (SMO) inhibitor approved for the treatment of locally advanced basal cell carcinoma and represents an increasingly important therapeutic alternative (36). While both agents target the Hedgehog signaling pathway and demonstrate comparable efficacy, sonidegib is characterized by a longer half-life and enhanced tissue penetration, which may allow for more flexible dosing strategies in clinical practice. The safety profiles of the two drugs are broadly similar, including muscle spasms, alopecia, dysgeusia, and weight loss; however, sonidegib has been associated with relatively lower rates of dysgeusia and gastrointestinal toxicity, alongside a higher incidence of creatine kinase elevation and myopathy. These differences may influence treatment selection, particularly in patients who develop intolerance to vismodegib (37).

Our findings provide the first pooled evidence focused specifically on periocular and orbital BCC, and while they parallel results from larger landmark trials, important distinctions must be noted. In our analysis, complete and partial response rates were 38.4 and 39.2%, respectively. These figures are broadly consistent with ERIVANCE (38) (overall response rate 43%) and STEVIE (39) (response rate ~60%) but should be interpreted cautiously, as both trials included patients with advanced BCC at any anatomical site rather than being restricted to the periocular region. The lower complete response rate in our review likely reflects the greater complexity of orbital disease, which is frequently T4 stage, recurrent after surgery or radiotherapy, and often associated with bone or orbital invasion. Similarly, we observed an exenteration rate of 28.1% following vismodegib, a substantial reduction compared with historical surgical series where orbital invasion nearly always necessitated exenteration. Safety outcomes were also comparable to those in broader cohorts, with nearly all patients (90.3%) developing adverse events and one-third (31.2%) discontinuing therapy due to intolerance (38, 39).

In comparison with the recent American Academy of Ophthalmology systematic review (40), which included 16 studies and highlighted complete response rates as high as 88% in individual cohorts with overall response rates up to 92%, our pooled estimates are more conservative, with 38.4% achieving complete response and 39.2% partial response and this high variability derived mainly from Ishai et al. 2020 (41) and Tiosano Et al 2023 (42) as they have more than half of population of all included studies and incidence of CR without including these 2 studies will go up to 45.5%. This discrepancy likely reflects differences in methodology, as the AAO review synthesized results largely qualitatively, whereas our study employed meta-analytic pooling that accounts for variability across cohorts. Both reviews, however, reached similar conclusions regarding the organ-preserving potential of vismodegib, with our pooled exenteration rate of 28.1% paralleling their reported range of 10–23%. Importantly, both analyses documented recurrence as a persistent challenge, with the AAO review noting relapses even after initial complete regression and molecular studies confirming residual tumor with resistance mutations, while our meta-analysis quantified recurrence at 17.4% after treatment discontinuation. Adverse events were also similarly reported as nearly universal, with the AAO review citing 95–100% incidence and discontinuation in up to 62% of patients, which is consistent with our pooled estimates of 90.3 and 31.2%, respectively (40).

Beyond clinical response rates, the patient experience with vismodegib is profoundly shaped by its adverse event profile. Alopecia, muscle spasms, and dysgeusia, while not life-threatening, substantially affect daily functioning, self-image, and social well-being, often driving discontinuation despite ongoing tumor control (21, 43). Quality-of-life considerations are therefore central to shared decision-making, particularly in elderly patients where treatment goals may prioritize comfort and independence over maximal oncologic control (44). Recurrence after discontinuation also remains a key challenge. Although some patients respond again upon rechallenge with vismodegib, others develop resistance driven by secondary SMO mutations or activation of non-canonical Hedgehog pathways (45, 46).

While vismodegib remains the most widely used Hedgehog pathway inhibitor, sonidegib has also demonstrated activity in advanced BCC, with similar efficacy but a slightly different toxicity profile that may make it preferable for select patients (47, 48). Recent advances point toward combination approaches, such as pairing Hedgehog inhibitors with immune checkpoint blockades or targeted radiotherapy, as potential avenues to improve depth and durability of response (49).

### 4.1 Future directions

The findings of this meta-analysis highlight several priorities for future research. First, prospective multicenter registries with standardized definitions of tumor stage, treatment duration, and response assessment are needed to reduce heterogeneity and enable more precise outcome estimation. Second, studies evaluating optimal treatment duration, intermittent dosing strategies, and toxicity mitigation protocols may improve long-term tolerability without compromising tumor control. Third, comparative effectiveness studies assessing vismodegib against alternative Hedgehog pathway inhibitors, such as sonidegib, or evaluating combination strategies with surgery, radiotherapy, or immunotherapy, may help clarify the optimal sequencing of therapies in advanced periocular and orbital disease. Finally, translational research investigating molecular predictors of response, resistance mechanisms, and tumor microenvironment characteristics could enable biomarker-driven patient selection and more individualized treatment strategies.

Although this meta-analysis provides a comprehensive synthesis of available evidence, the findings should be interpreted with caution. All included studies were observational in design, and sample sizes varied considerably across cohorts, which may limit the robustness and external validity of the pooled estimates. As such, the results should be considered hypothesis-generating and supportive of emerging clinical evidence rather than definitive. Further high-quality prospective studies are required to validate these findings and to establish standardized treatment recommendations.

This review has several strengths that enhance the robustness of its findings. It represents the largest synthesis to date of vismodegib outcomes specifically in periocular and orbital BCC, incorporating data from diverse geographic regions and including both prospective and retrospective cohorts. The use of standardized tools (NOS and JBI) ensured rigorous assessment of study quality, and the application of quantitative meta-analysis allowed pooled estimates across key efficacy and safety outcomes. Nonetheless, important limitations must be acknowledged. Most included studies were retrospective, with inherent risk of selection bias and incomplete follow-up. Considerable heterogeneity was observed across several outcomes, likely reflecting variability in study populations, definitions of response, follow-up duration, and prior treatments. Furthermore, vismodegib was sometimes combined with other modalities, making it difficult to attribute outcomes exclusively to the drug. These limitations underscore the need for future research in the form of large, multicenter prospective studies or registries with standardized outcome reporting. Randomized controlled trials comparing vismodegib with surgery or radiotherapy in borderline operable tumors could clarify its optimal role, while translational studies investigating predictive biomarkers may help identify patients most likely to benefit and tolerate therapy.

## 5 Conclusion

This systematic review and meta-analysis synthesize the currently available clinical evidence and demonstrates that vismodegib is an effective therapeutic option for locally advanced periocular and orbital basal cell carcinoma, achieving substantial rates of tumor regression and contributing to organ preservation in selected patients. However, frequent adverse events, treatment discontinuation, and recurrence after cessation remain significant clinical challenges.

The findings of this analysis underscore several priorities for future evidence generation, including prospective multicenter registries with standardized outcome reporting, studies evaluating optimal treatment duration and toxicity mitigation strategies, and comparative effectiveness investigations assessing Hedgehog pathway inhibitors within multimodal treatment frameworks. In addition, translational research aimed at identifying predictive biomarkers of response and resistance may enable more individualized therapeutic selection and improve long-term disease control. Until such data become available, careful patient selection, close toxicity monitoring, and multidisciplinary treatment planning remain essential to optimize clinical outcomes.

## Data Availability

The original contributions presented in the study are included in the article/supplementary material, further inquiries can be directed to the corresponding author.
